# Increased tibial tubercle torsion is associated with advanced lateral patellofemoral osteoarthritis

**DOI:** 10.1002/jeo2.70637

**Published:** 2026-01-19

**Authors:** Maksym Polt, Lukas Jud, Sandro Hodel, Andreas Flury, Benjamin Fritz, Lazaros Vlachopoulos, Sandro F. Fucentese

**Affiliations:** ^1^ Department of Orthopaedics Balgrist University Hospital Zurich Switzerland

**Keywords:** femoral antetorsion, hip–knee–ankle angle, lateral patellofemoral osteoarthritis, patellofemoral osteoarthritis, tibial tubercle torsion

## Abstract

**Purpose:**

To investigate whether the emerging anatomical parameters of the patellofemoral (PF) joint, including tibial tubercle torsion (TT torsion), the sagittal tibial tuberosity–trochlear groove (sTT–TG) distance, the tibial tuberosity rotational angle (TT–RA), the tibiofemoral rotation and tibial tuberosity lateralisation (TT lateralisation), are associated with advanced lateral patellofemoral osteoarthritis (PFOA).

**Methods:**

This retrospective study analysed patients from a preoperative total knee arthroplasty workup database. PF cartilage lesions of International Cartilage Repair Society Grade 3 or higher were defined as advanced. TT torsion, sTT–TG, TT–RA and TT lateralisation were assessed based on computed tomography imaging. Additionally, tibiofemoral rotation, tibial tuberosity–trochlear groove distance, Caton–Deschamps index, femoral antetorsion, tibial torsion and the hip–knee–ankle (HKA) angle were also included in the analysis. Binary logistic regression analysis and Mann–Whitney *U* test were used to examine associations between the advanced PFOA and the measured anatomical parameters.

**Results:**

Fifty‐two knees/patients (35 females and 17 males) could be included: 20 with advanced lateral PFOA, 13 with advanced medial PFOA and 19 without advanced PFOA. Femoral antetorsion (odds ratio [OR] = 1.094, 95% confidence interval [CI] = 1.029–1.164; *p* = 0.004) and valgus HKA angle (OR = 1.152, 95% CI = 1.066–1.247; *p* < 0.001) met the Bonferroni‐adjusted threshold (*p* < 0.005) for association with lateral PFOA. TT torsion was significant at the 0.05 level but not after the Bonferroni adjustment (OR = 1.138, 95% CI = 1.027–1.260; *p* = 0.013). Analysing isolated lateral vs. medial PFOA, higher TT torsion and femoral torsion, as well as valgus HKA angle, exhibited a Bonferroni‐adjusted statistically significant correlation with advanced lateral PFOA (*p* = 0.003, *p* < 0.001 and *p* < 0.002, respectively).

**Conclusion:**

TT torsion is associated with advanced lateral PFOA and may represent an additional risk factor. This finding underscores the substantial role of rotational malalignment in the pathogenesis of lateral PFOA. Recognising the role of TT torsion and its intercorrelation with the HKA angle and femoral antetorsion may refine clinical assessment of PF conditions and inform surgical strategies.

**Level of Evidence:**

Level III.

**Abbreviations**
BMI: body mass index
CDI: Caton–Deschamps index
CT: computed tomography
HKA: hip–knee–ankle
ICCs: interclass correlation coefficients
ICRS: International Cartilage Repair Society
MRI: magnetic resonance imaging
OA: osteoarthritis
OR: odds ratio
PF: patellofemoral
PFJ: patellofemoral joint
PFOA: patellofemoral osteoarthritis
sTT‐TG: sagittal tibial tuberosity–trochlear groove
TKA: total knee arthroplasty
TT lateralisation: tibial tuberosity lateralisation
TT–RA: tibial tuberosity rotational angle
TT–TG: tibial tuberosity–trochlear groove
TT torsion: tibial tubercle torsion

## INTRODUCTION

Patellofemoral osteoarthritis (PFOA) is prevalent among individuals experiencing knee symptoms and often represents an early manifestation of knee osteoarthritis (OA) that precedes involvement of the tibiofemoral joint [9, 16, 17, 28]. In isolation, arthritic changes were found in the patellofemoral (PF) compartment in 13.6% of women and 15.4% of men aged over 60 [8]. Lateral PFOA is the most common form and is associated with biomechanical abnormalities such as PF dysplasia, increased tibial tuberosity–trochlear groove (TT–TG) distance and valgus malalignment of the leg, expressed as a valgus hip–knee–ankle (HKA) angle [1, 6, 11, 15, 18, 29]. Other risk factors include a high‐positioned patella [29], recurrent lateral patella dislocation [32], increased femoral anteversion [13], sex, body mass index (BMI), and a history of trauma [5].

Due to the complexity of the PF joint, different novel anatomical parameters with possible influence on the biomechanics have been described. These include tibial tubercle torsion (TT torsion) [4], sagittal TT–TG (sTT–TG) distance [25], tibial tuberosity rotational angle (TT–RA) [10], tibiofemoral rotation [10], and tibial tuberosity lateralisation (TT lateralisation) [35]. Except for the sTT–TG distance, which recent studies have linked to patellar cartilage lesions or degenerative changes [21, 25, 30], factors such as TT–RA [22], tibiofemoral rotation [10], TT lateralisation [35], and TT torsion [4] have not yet been associated with PFOA. At the same time, increased TT torsion has been identified as a potentially relevant risk factor for PF instability [4, 20]. We therefore assumed that increased TT torsion might also be associated with lateral PFOA.

Therefore, the objective of this study was to investigate whether TT torsion, sTT–TG distance, TT‐RA, tibiofemoral rotation, and TT lateralisation are associated with lateral PFOA. We hypothesised that increased TT torsion, leading to increased stress on the lateral side of the PF joint, would demonstrate the strongest association with lateral PFOA.

## MATERIALS AND METHODS

**Figure 1:**
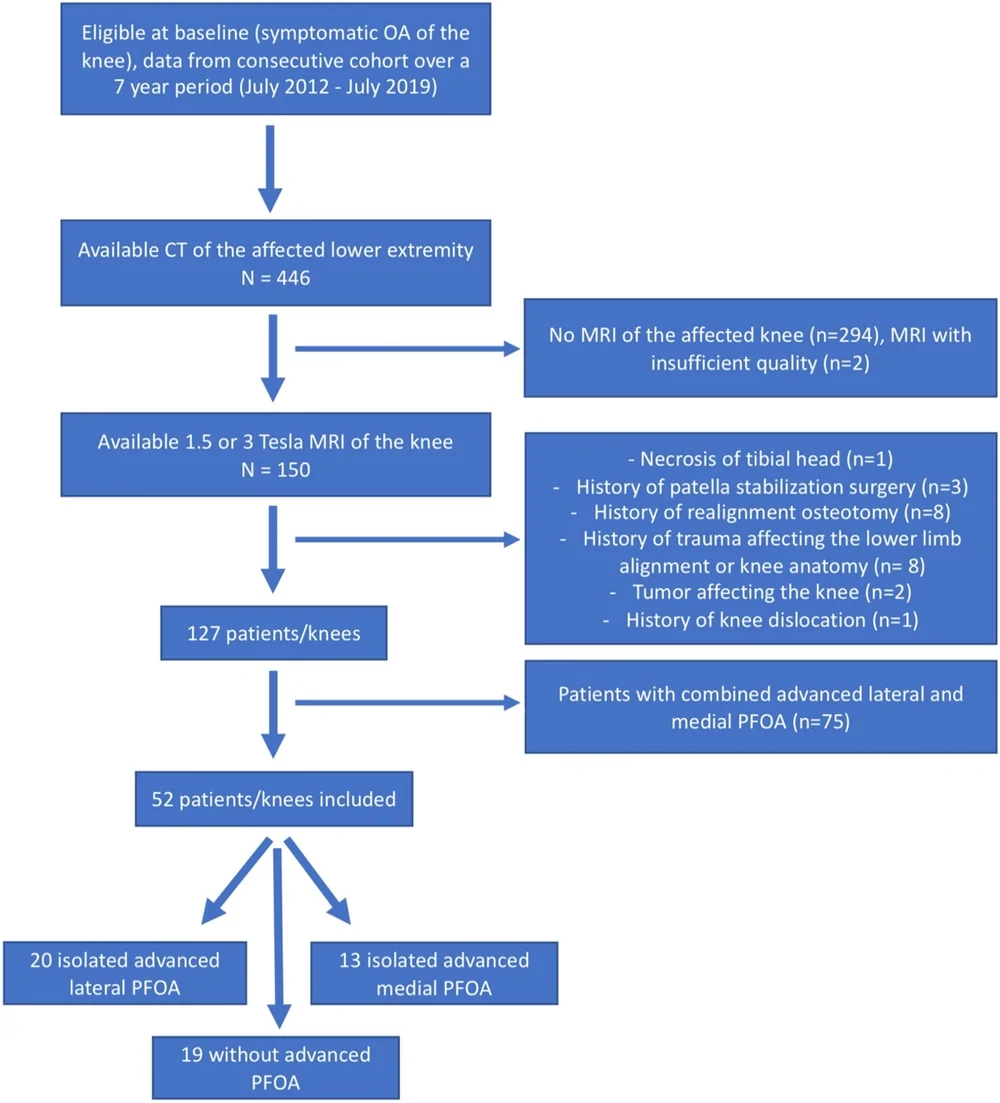
Study population flowchart. CT, computed tomography; MRI, magnetic resonance imaging; OA, osteoarthritis; PFOA, patellofemoral osteoarthritis.

This retrospective cohort study utilised an existing institutional database previously employed in the study by Flury et al. [13]. The database comprises patients who underwent preoperative workup for total knee arthroplasty (TKA) due to symptomatic OA involving any compartment of the knee between 2012 and 2019. Ethical approval for the use of this data set was granted by the Zurich Cantonal Ethics Commission (KEK 2020‐00809), and all patients had provided written informed consent allowing the use of their anonymized data for clinical research purposes. We included patients with available computed tomography (CT) and magnetic resonance imaging (MRI) (1.5 or 3 T) of the affected knee (Figure 1). The exclusion criteria were the following: insufficient MRI quality due to motion artefacts, history of trauma, surgery, osteonecrosis, or tumour affecting limb alignment or knee anatomy (Figure 1). Following the identification of patients who had undergone both MRI for diagnostic evaluation and CT scanning for preoperative planning, and after applying the exclusion criteria, a cohort of 127 patients was obtained for further analysis of the exact location and severity of the PF cartilage damage (Figure 1). The PF cartilage condition had been assessed by a fellowship‐trained musculoskeletal radiologist for an earlier investigation [13], using the International Cartilage Repair Society (ICRS) classification system: Grade 1 for superficial cartilage changes; Grade 2 for lesions extending to less than 50% of the cartilage depth; Grade 3 for lesions involving more than 50% of the cartilage depth; and Grade 4 for complete cartilage loss. Cartilage lesions of Grade 3 or higher were defined as advanced [13]. Anatomically, lateral PFOA (lateral patella facet, lateral trochlea facet, or both) and medial PFOA (medial patella facet, medial trochlea facet, or both) were distinguished. To enable a targeted assessment of the mechanical factors contributing to compartmental cartilage degeneration, patients with combined advanced lateral and medial PFOA were excluded, as shown in Figure 1. Of the remaining 52 knees, 20 had isolated advanced lateral PFOA, and 13 had isolated advanced medial PFOA. In 19 knees, no advanced PFOA was present in either compartment. Thus, this group served as the control group for the subsequent statistical analyses.

### Radiologic imaging assessment

**Figure 2:**
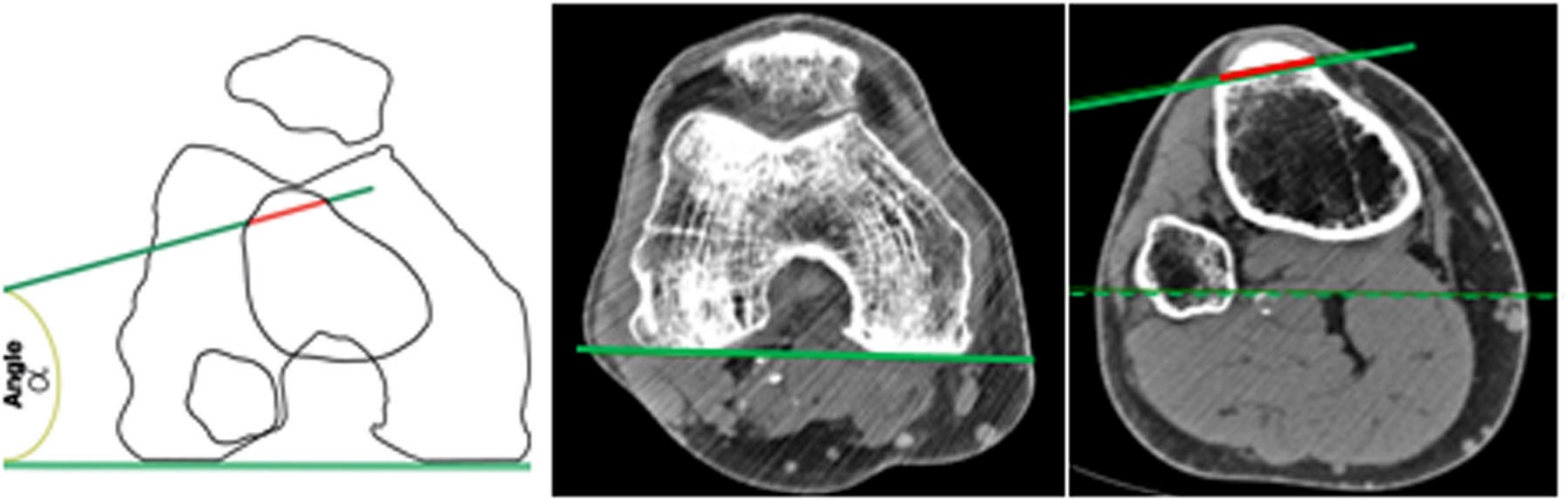
TT torsion is measured as the angle between the tangent of the posterior femoral condyles and a line connecting the most medial and lateral insertions of the patellar tendon on the TT [7]. TT, tibial tubercle.

**Figure 3:**
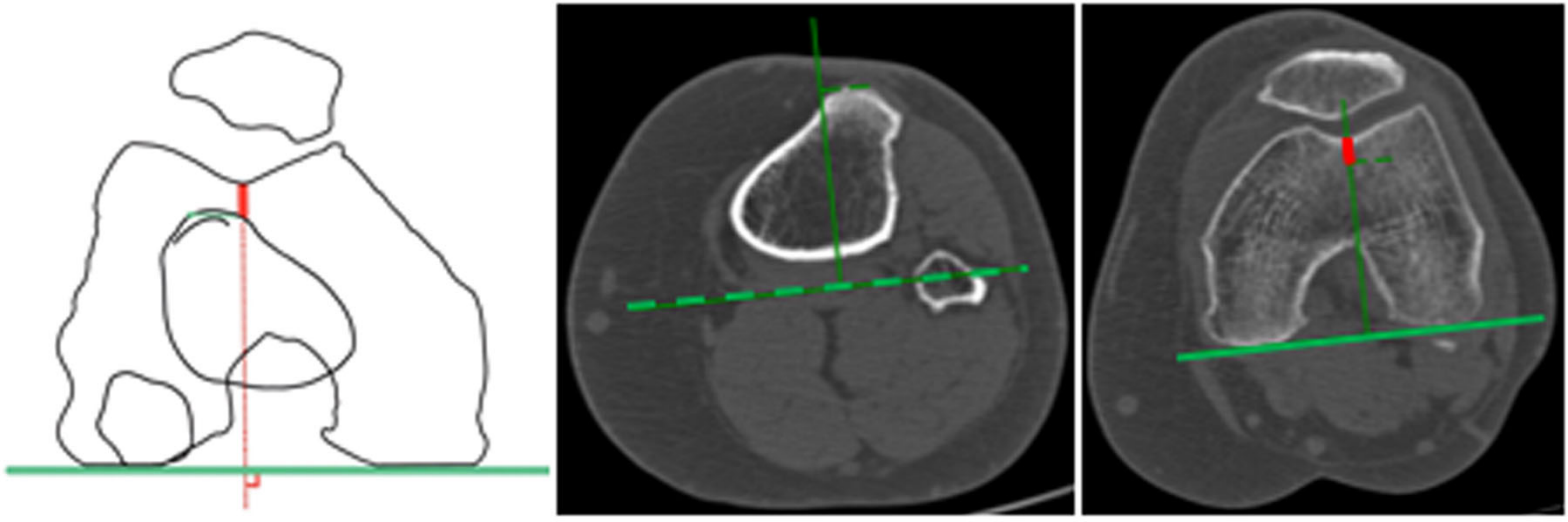
The sTT–TG distance is measured as the perpendicular distance between the point at the nadir of the trochlear groove and a point at the anterior aspect of the TT, relative to the posterior condylar axis [8]. sTT–TG, sagittal tibial tuberosity–trochlear groove; TT, tibial tubercle.

**Figure 4:**
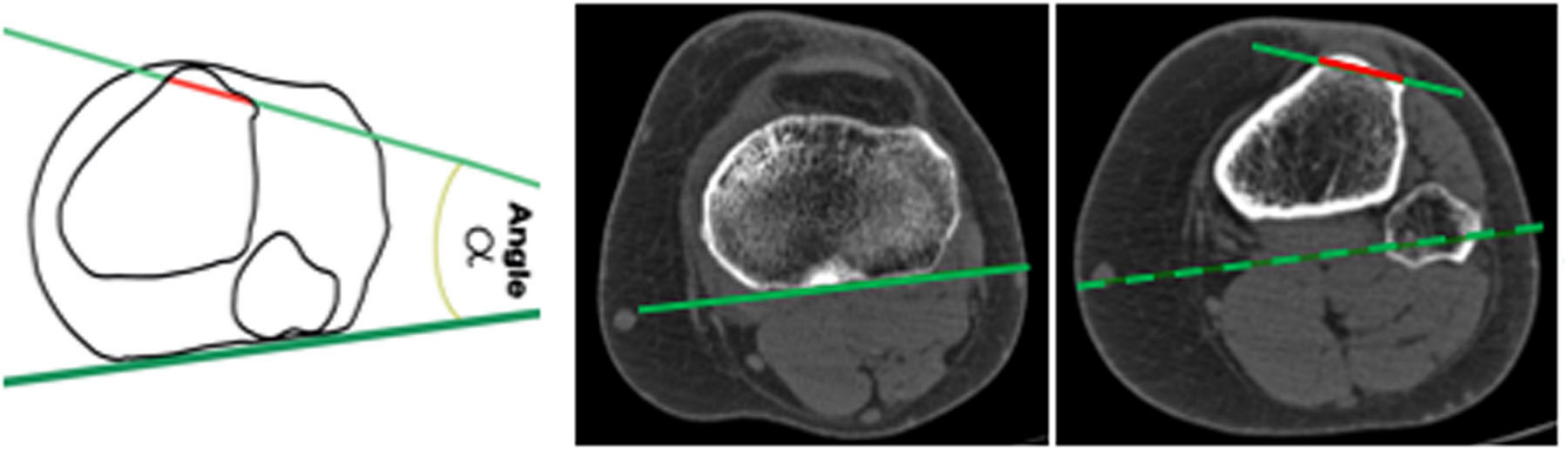
TT–RA is measured as the angle between the dorsal tibial condylar line and the same patellar tendon line used for TT torsion [9]. TT, tibial tubercle; TT–RA, tibial tuberosity rotational angle.

CT scans of the knee were acquired, and transverse reconstructions with a slice thickness of 1 mm were used for torsion measurements. All assessments were performed on a standardised picture archiving and communication system workstation using a standardised technique by a senior orthopaedic resident, as detailed in the subsequent section. The investigator was blinded to the PF cartilage condition. In line with clinical relevance, outcome variables are reported to one decimal place. The following parameters were assessed based on CT imaging, as illustrated in Figures 2, 3, 4. TT torsion was measured as the angle between the tangent of the posterior femoral condyles and a line connecting the most medial and lateral insertions of the patellar tendon on the TT [4]. The sTT–TG distance was measured as the perpendicular distance between the point at the nadir of the trochlear groove (on the anterior surface of the trochlear cartilage) and a point at the anterior aspect of the TT, relative to the posterior condylar axis [25]. The TT–RA was measured as the angle between the dorsal tibial condylar tangent and the same patellar tendon line used for TT torsion [22]. Tibiofemoral rotation was measured as the angle between the tangent of the posterior femoral condyles and the tangent of the dorsal tibial plateau condyles [10]. Finally, TT lateralisation was measured as the lateral position of the TT as a percentage of the total tibial plateau width, determined by measuring the medial‐to‐lateral distance from the most anterior point of the TT to the medial edge of the tibial plateau, using lines perpendicular to the dorsal tibial plateau condylar tangent [35].

Further relevant knee joint parameters, previously assessed by Flury et al. in their study on lateral retropatellar cartilage degeneration [13], were included in the statistical analysis. These already measured parameters comprised femoral antetorsion, tibial torsion,‐ and the frontal mechanical axis. The Caton–Deschamps index (CDI) and the TT–TG distance were measured separately [3, 15] and likewise included in the statistical analysis.

### Statistical analysis

Descriptive statistics were performed to summarise the characteristics of the study population, including sex, age and BMI. All anatomical parameters of interest were reported as mean values with standard deviations for continuous variables. For categorical variables, data are presented as absolute frequencies and percentages. All statistical analyses were performed using SPSS (version 25.0; IBM Corp.). Statistical significance was defined as a *p*‐value < 0.05. Spearman's rank correlation test was performed to explore general trends of association between the anatomical parameters and the presence of advanced PFOA. To control the risk of false positives, the Bonferroni correction was performed. Given that 10 anatomical parameters were tested for correlation with medial and lateral PFOA, the corrected significance threshold was set at *p* < 0.005 (0.05/10). Accordingly, *p*‐values < 0.005 were considered statistically significant after Bonferroni adjustment, while results with 0.005 ≤ *p* < 0.05 are reported as nominal findings and interpreted with caution. A binary logistic regression was performed to examine associations between the presence of advanced PFOA (medial or lateral) and the measured anatomical parameters, with advanced status defined by dichotomised ICRS grades (0–2 vs. 3–4). The model yielded odds ratios (ORs) with 95% confidence intervals. Finally, a subgroup analysis was performed using the Mann–Whitney *U* test, in which the measured anatomical parameters were compared between isolated medial and isolated lateral PFOA.

## RESULTS

**Figure 5:**
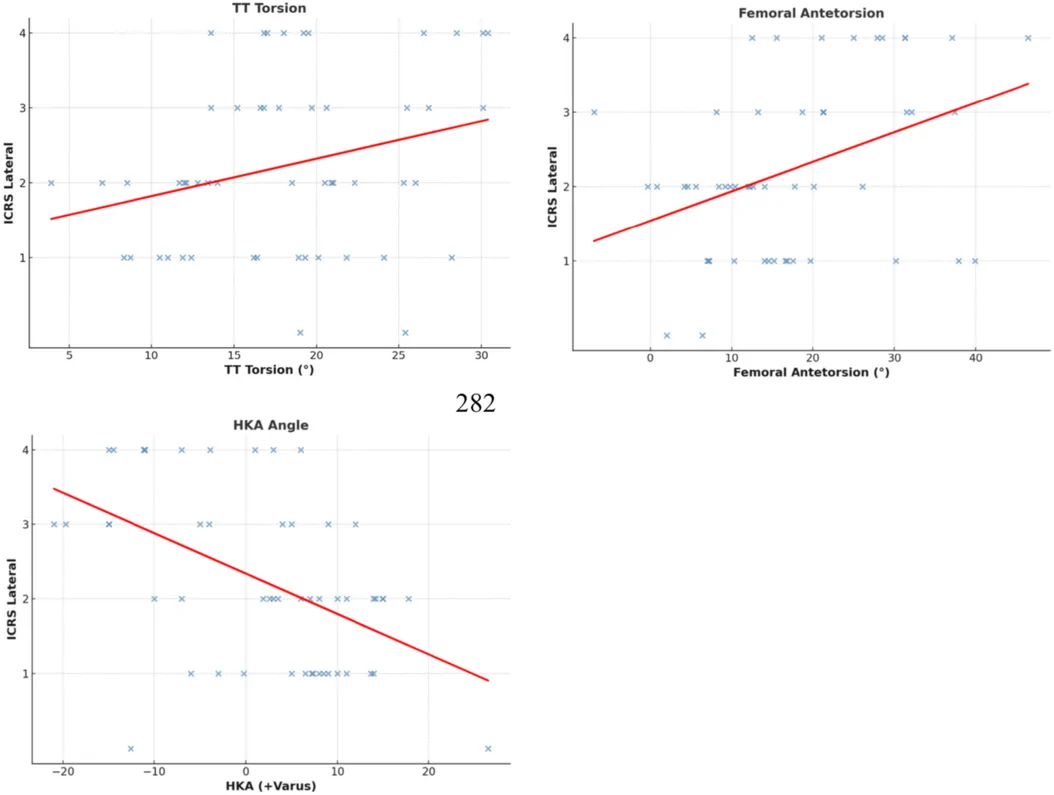
Significant linear correlation between lateral PFOA and increased TT torsion, increased femoral antetorsion, and valgus HKA angle. HKA, hip–knee–ankle; ICRS, International Cartilage Repair Society; PFOA, patellofemoral osteoarthritis; TT, tibial tubercle.

Fifty‐two knees in 52 patients (35 females and 17 males) were included in the analysis. Twenty patients had isolated advanced lateral PFOA (ICRS Grade 3–4), 13 had isolated advanced medial PFOA, and 19 had no advanced PFOA in either compartment. Descriptive statistics of demographic and anatomical parameters of interest are summarised in Table 1. Exploring general associations and taking the sign of the correlation coefficient (CC) into account, only higher femoral antetorsion (positive CC) and greater HKA angle in the direction of valgus (negative CC, given valgus < 0) remained statistically significant for lateral PFOA after Bonferroni correction (*p* < 0.005), as demonstrated in Table 2. Increased TT torsion did not reach the adjusted threshold but was associated with lateral PFOA at the 0.05 level (*p* = 0.049) and inversely associated with medial PFOA (*p* = 0.035). Figure 5 illustrates the association between the discussed parameters and the lateral PFOA.

**Table 1:** Patient demographics and radiological parameters.

Variable	Value
Patients included	52
Sex	35 females/17 males
Age (years)	64.7 ± 8.7
BMI	32.0 ± 6.8
Lateral PFOA (ICRS 3–4)	20
Medial PFOA (ICRS 3–4)	13
No advanced PFOA (ICRS < 3)	19
Femoral antetorsion (°)	17.4 ± 11.6
Tibial torsion (°)	22.5 ± 9.8
HKA (°)	2.0 ± 10.8
CDI	1.0 ± 0.2
TT–TG (mm)	15.2 ± 4.3
sTT–TG (mm)	4.8 ± 7.9
TT torsion (°)	18.2 ± 6.5
TT‐RA (°)	17.7 ± 5.4
Tibiofemoral rotation (°)	0.9 ± 4.2
TT lateralisation	0.7 ± 0.04

*Note*: Continuous variables are presented as means ± standard deviation (SD).

Abbreviations: BMI, body mass index; CDI, Caton–Deschamps index; HKA, hip–knee–ankle; ICRS, International Cartilage Repair Society; PFOA, patellofemoral osteoarthritis; sTT–TG, sagittal tibial tuberosity‐trochlear groove; TT, tibial tubercle; TT‐RA, tibial tuberosity rotational angle; TT‐TG, tibial tuberosity‐trochlear groove.

**Table 2:** Spearman's rank correlation test.

Parameter	*p*‐value	CC	95% CI
**(a) Correlation with lateral PFOA**			
TT torsion (°)	0.049	0.275	[−0,007 to 0.515]
Femoral antetorsion (°)	0.004	0.388	[0.121–0.603]
HKA (°)	<0.001	−0.510	[−0.693 to −0.265]
**(b) Correlation with medial PFOA**			
TT torsion (°)	0.035	−0.293	[−0,530 to −0.14]
Femoral antetorsion (°)	0.021	−0.319	[−0.551 to −0.043]
HKA (°)	0.196	0.184	[−0.105 to 0.444]

*Note*: TT torsion, femoral antetorsion, and HKA were the only parameters showing *p*‐values < 0.05 in association with advanced lateral (a) or medial (b) PFOA. Femoral antetorsion and HKA angle remained statistically significant for lateral PFOA after Bonferroni correction (*p* < 0.005).

Abbreviations: CC, correlation coefficient; CI, confidence interval; HKA, hip–knee–ankle; PFOA, patellofemoral osteoarthritis; TT, tibial tubercle.

Binary logistic regression identified two parameters that met the Bonferroni‐adjusted threshold (*p* < 0.005) for association with lateral PFOA: femoral antetorsion (per 1°: OR = 1.094, 95% CI = 1.029–1.164; *p* = 0.004) and HKA per 1° towards valgus (OR = 1.152, 95% CI = 1.066–1.247; *p* < 0.001), indicating greater valgus alignment is associated with lateral PFOA. TT torsion did not meet the adjusted threshold but was statistically significant at the 0.05 level (per 1°: OR = 1.138, 95% CI = 1.027–1.260; *p* = 0.013), and is therefore highlighted as a key association in this cohort (Table 3). No significant correlation was found for tibial torsion, CDI, TT–TG, sTT–TG, TT–RA, tibiofemoral rotation, or TT lateralisation.

**Table 3:** Binary logistic regression models for advanced lateral (a) and medial (b) PFOA.

(a) Binary logistic regression model for advanced lateral PFOA			
Parameter	Odds ratio (OR)	*p*‐value	95% CI
TT torsion (°)	1.138	0.013	[1.027–1.260]
Femoral antetorsion (°)	1.094	0.004	[1.029–1.164]
HKA (°)	1.152	<0.001	[1.066–1.247]

(b) Binary logistic regression model for advanced medial PFOA			
Parameter	OR	*p*‐value	95% CI
TT torsion (°)	0.880	0.031	[0.783–0.988]
Femoral antetorsion (°)	0.906	0.016	[0.837–0.982]
HKA (°)	0.943	0.089	[0.880–1.009]

*Note*: TT torsion, femoral antetorsion, and HKA angle showed significant ORs for lateral PFOA (*p* < 0.05); for medial PFOA, only TT torsion and femoral antetorsion. After Bonferroni correction (*p* = 0.005), only femoral antetorsion and HKA remained significant, and only for lateral PFOA. HKA was coded valgus <0 and varus >0; ORs (and CIs) are reported per 1° towards valgus.

Abbreviations: CI, confidence interval; HKA, hip–knee–ankle; PFOA, patellofemoral osteoarthritis; TT, tibial tubercle.

The subgroup analysis comparing patients with isolated advanced lateral versus isolated advanced medial PFOA revealed that a higher TT torsion, higher femoral antetorsion, and HKA angle in the direction of valgus exhibited a Bonferroni‐adjusted statistically significant correlation with advanced lateral PFOA (*p* = 0.003, *p* < 0.001 and *p* < 0.002, respectively) (Table 4). For advanced medial PFOA, the same parameters differed in the opposite direction.

**Table 4:** Subgroup analysis of isolated advanced lateral versus medial PFOA (Mann–Whitney *U* test).

Parameter		Lateral PFOA	Medial PFOA
TT torsion (°)	*p*‐value	0.003	<0.001
	Mean ± SD	21.1 ± 5.8	14.6 ± 5.8
Femoral antetorsion (°)	*p*‐value	<0.001	<0.001
	Mean ± SD	23.7 ± 11.9	10.1 ± 4.7
HKA (°)	*p*‐value	0.002	<0.001
	Mean ± SD	−5.7 ± 9.9	6.5 ± 7.1

*Note*: TT torsion, femoral antetorsion, and HKA angle exhibited a Bonferroni‐adjusted statistically significant correlation. They are presented along with their mean values and standard deviations (SDs).

Abbreviations: HKA, hip–knee–ankle; PFOA, patellofemoral osteoarthritis; TT, tibial tubercle.

## DISCUSSION

The most important finding of this study is the identification of increased TT torsion as a possible additional risk factor for lateral PFOA. This is the first study to demonstrate a strong association between TT torsion and lateral PFOA. Additionally, a valgus HKA angle and increased femoral antetorsion were also associated with lateral PFOA. These two parameters are already known to be linked with lateral PFOA [11, 13, 19], and our findings confirm their relevance in this context.

PFOA is highly prevalent, affecting nearly 40% of symptomatic knees and appearing as an isolated compartmental disease in 10%–20% of cases [8, 24]. The patellofemoral joint (PFJ) is biomechanically complex: multiple static and dynamic stabilisers control patellar tracking in all three planes, so that even a single disturbance can distort contact mechanics and concentrate load primarily on the lateral facet [1]. Research interest in the causes of PFOA has therefore increased, examining malalignment, PF dysplasia, PF instability, trauma, and inflammatory arthritis [1, 14, 23]. Rotational malalignment has also gained attention: excessive femoral antetorsion and external tibial torsion are now linked to anterior knee pain, higher PF contact pressures, and progressive cartilage degeneration [12, 13, 26, 33, 36]. Yet the torsional orientation of the tibial tuberosity, TT torsion, has remained unexplored as a possible contributor to PFOA, an issue addressed by the present study.

This work is the first to show a statistically significant association between TT torsion and PFOA, although the result did not meet the Bonferroni‐adjusted significance threshold. Thus, our study is extending a concept that until now had been limited to patellar instability. Chassaing et al. found that every 10° increase in TT torsion multiplied the risk of instability 11‐fold and discriminated better than the TT–TG distance [4], while Jud et al. reported an OR of 55 for instability at a CT cut‐off of 17.7° [20]. In comparison, the mean TT torsion in the investigated cohort was 18.2 ± 6.5°. After adjusting for anatomic and biomechanical covariates in our cohort, each additional degree of TT torsion raised the odds of lateral PFOA by 14% (OR = 1.138; 95% CI = 1.027–1.260). Cadaveric pressure‐film studies support this mechanism: external tibial rotation, effectively mimicking tubercle rotation, selectively increases stress on the ipsilateral patellar facet and accelerates cartilage wear [27]. Although no prior study has directly examined TT torsion in relation to retropatellar contact stress or PFOA development, the measurement itself was shown to be reliable (interclass correlation coefficient [ICC] = 0.88) [4, 20]. By establishing a connection with lateral PFOA, our findings position TT torsion as an additional, quantifiable structural driver of PF degeneration. This finding could have major implications for surgical strategy, for example, integrating preoperative assessment of TT torsion into routine work‐up, or considering a derotational TT osteotomy in symptomatic patients with excessive TT torsion.

A valgus HKA angle and increased femoral antetorsion complemented TT torsion as predictors of lateral PFOA. These findings are supported by the 292‐knee radiographic study by Elahi et al., where 57% of lateral PFOA knees were valgus versus only 26% of medial PFOA knees [11]. More recently, it was demonstrated that the contact centre of the patellar facet shifts progressively laterally as the valgus HKA angle increases [19]. Similarly, Flury et al. reported that femoral antetorsion >20° tripled the odds of ICRS Grade 3–4 cartilage lesions on the lateral facet, with the effect even stronger in valgus knees [13]. Together, these data suggest that both valgus alignment and excessive femoral antetorsion heighten lateral PFJ stress, predisposing the lateral cartilage to degeneration over time. Our findings concerning TT torsion point to a common mechanism: increased TT torsion, valgus HKA angle, and greater femoral antetorsion appear to work together to push the patella outward and overload the lateral PF cartilage.

Further parameters analysed, such as sTT–TG, tibial torsion, CDI, TT–TG, TT–RA, tibiofemoral rotation, and TT lateralisation, demonstrated no significant correlation with advanced isolated lateral or medial PFOA in our cohort. Among these parameters, it is important to discuss sTT–TG, as emerging evidence suggests a link between sTT–TG and PFOA. Lansdown et al., who introduced this parameter, reported a markedly more posterior TT in patients undergoing cartilage repair than in matched asymptomatic controls [25]. Kaplan et al. later confirmed that a more posterior TT correlates with increased risk for PF chondral lesions [21]. In a large, multi‐centre cohort using deep learning analysis of MRI scans, Namiri et al. reported that a more posteriorly positioned TT was significantly associated with the presence of PFOA [30]. In the latest study on sTT–TG, Bi et al. found that patients with a more posteriorly positioned TT tended to have larger chondral defects in the PFJ [2], underlining the importance of this new parameter. The results of our study diverge from the discussed findings. A possible reason for that is a different study design: in contrast to the mentioned studies, which analysed mixed PF lesions, we focused on isolated advanced medial or lateral defects. Under this compartment‐specific evaluation, rotational stress from TT torsion likely dominates load distribution, making sagittal malalignment (sTT–TG) less influential and thus non‐correlative in our data set. In general, our results emphasise the importance of considering the overall tibia–femur construct in all three planes for understanding patellar tracking and PF pathologies. Anatomical TT parameters that relate exclusively to the tibia appear to play a subordinate role and thus did not show statistical significance in our analysis. Regarding CDI and TT–TG, both are well‐known risk factors for PFOA [7, 15, 31, 34]. Our cohort showed values near normal (1.0 ± 0.2 and 15.4 ± 4.7 mm, respectively), and thus we could not detect any association with PFOA.

Our study has some limitations. First, because of multiple comparisons in a relatively small cohort, we applied a Bonferroni correction (*p* = 0.005). Under this threshold, the OR for TT torsion was statistically significant at *p* < 0.05 but not after Bonferroni adjustment. Therefore, the TT torsion OR must be interpreted with caution. At the same time, we deliberately restricted the analysis to cases with isolated advanced lateral or medial PFOA, aiming to better evaluate the specific mechanical factors contributing to compartmental cartilage degeneration. Second, our cohort included only elderly patients with end‐stage disease. Restricting the sample to TKA candidates may be seen as a selection bias and could lead to additional confounders, limiting the generalisability of our findings. Moreover, the unequal gender distribution (35 females and 17 males) may further contribute to this limitation. Third, the measurements were performed manually, and no ICCs were calculated. However, the measurement technique employed has previously been demonstrated to be both reproducible and reliable in earlier studies [4, 13, 20, 22, 25]. Finally, a retrospective study design is a further limitation. At the same time, it was the only feasible way to conduct this study. Larger prospective studies that include patients of different ages and include a control group without PFOA will be needed to validate and refine our observations, or even to obtain a dose–response estimate of how TT torsion influences compartment‐specific PFOA.

## CONCLUSION

Among both well‐established and emerging anatomical parameters, TT torsion was associated with advanced lateral PFOA and may represent an additional risk factor for it. This finding underscores the substantial role of rotational malalignment in the pathogenesis of lateral PFOA. Recognising the role of TT torsion and its intercorrelation with the HKA angle and femoral antetorsion may refine clinical assessment of patellofemoral conditions and inform surgical strategies.

## AUTHOR CONTRIBUTIONS

**Maksym Polt**: Conceptualisation; investigation; writing and editing of the manuscript. **Lukas Jud**: Conceptualisation; project administration; editing of the manuscript. **Sandro Hodel**: Methodology; statistical analysis. **Andreas Flury**: Investigation; formal analysis. **Benjamin Fritz**: Investigation; formal analysis. **Lazaros Vlachopoulos**: Project administration; formal analysis. **Sandro F. Fucentese**: Supervision; project administration; editing of the manuscript.

## CONFLICT OF INTEREST STATEMENT

The authors declare no conflicts of interest.

## ETHICS STATEMENT

Ethical approval for the use of this data set was granted by the Zurich Cantonal Ethics Commission (KEK 2020‐00809). All participants provided written informed consent to participate in the study and for their data to be used and published.

## Data Availability

The data sets used in this study are available from the corresponding author upon request.
